# Quality of life, quality of business, and destinations of recent graduates: fields of study matter

**DOI:** 10.1007/s00168-022-01117-x

**Published:** 2022-03-07

**Authors:** Arthur Grimes, Shaan Badenhorst, David C. Maré, Jacques Poot, Isabelle Sin

**Affiliations:** 1 Motu Economic and Public Policy Research, Wellington, New Zealand; 2 Victoria University of Wellington, Wellington, New Zealand; 3 Social Wellbeing Agency, Wellington, New Zealand; 4 Vrije Universiteit Amsterdam, Amsterdam, The Netherlands; 5 University of Waikato, Hamilton, New Zealand

**Keywords:** I23, J24, J61, R23, R58, Z13

## Abstract

One of the main challenges facing non-metropolitan regions is the attraction and retention of highly-educated young people. A loss of the brightest can lead to reduced business creation, innovation, growth and community well-being in such regions. We use rich longitudinal microdata from New Zealand to analyse the determinants and geography of the choice of destination of recent university and polytechnic graduates 2 years and 4 years after graduation. Rather than considering a range of location-specific consumption and production amenities, we assume spatial equilibrium and calculate, following Chen and Rosenthal (J Urban Econ 64:519–537, 2008), ‘quality of life’ and ‘quality of business’ indicators for urban areas that encompass all amenities that are utility and/or productivity enhancing (or reducing, in the case of disamenities). Specifically, we test whether students locate in places that are regarded as good to live or good to do business; and how this differs by field of study. Our estimates are conditional on students’ prior school (home) location and the location of their higher education institution. We find that graduates are attracted to locate in urban places that have high quality production amenities. High quality consumption amenities have heterogeneous effects on the location choice of students. Creative arts and commerce graduates are relatively more likely to locate in places that are attractive to business, consistent with a symbiosis between bohemians and business. Decision makers can leverage their existing local strengths, in terms of production and/or consumption amenities, to act as drawcards for, or to retain, recent graduates in specific fields.

## Introduction

A highly-educated population is one of the key drivers of local growth and prosperity. One of the main challenges facing non-metropolitan regions is therefore the attraction and retention of tertiary (university and polytechnic) educated graduates. A loss of the brightest can lead to reduced business creation, innovation, growth and community well-being. When there is no local university or polytechnic, regions will lose at least for some years their youth who seek a tertiary education.^1^ The chance of students returning upon graduation and the chance of attracting other graduates will depend on a range of ‘pull factors’, student characteristics, and where the tertiary education was undertaken. Some students may return to their home locality upon graduation, but others are likely to find work in the city in which the higher education institute (HEI) is located. Alternatively, graduates may move to another large city, or go abroad. The presence of an HEI, and especially a university, can itself contribute to population and employment growth in a region (Apatov and Grimes 2019).

We analyse a young person’s choice of work location during their first years after graduation, given the location of their tertiary education and the location of their prior schooling (i.e. home location). Our main interest is in the determinants of the location choice of where to work. Policymakers in smaller settlements frequently bemoan the loss of their brightest young people.^2^ We investigate whether there are ‘pull factors’ that could encourage the graduates to return to such areas.

Location choices by workers, and by firms, are driven by many individual-specific and location-specific factors. The available (dis)amenities data on cities and regions are unlikely to capture all relevant location features adequately. We therefore follow the approach of Chen and Rosenthal (2008) who calculate indicators of ‘quality of life’ (QL) and ‘quality of business’ (QB) for urban areas under the assumption that, in spatial equilibrium, local wages and rents reflect everything that matters locally for the utility of workers and for profits of firms. Spatial variation in wages and prices is then due to spatial variation in location-fixed amenities that impact on utility (such as a pleasant climate) or on profitability (such as good infrastructure). Following Roback (1982, 1988), it can be shown that a local increase in consumption amenities will lead to higher rents and lower wages, while greater production amenities will lead to higher rents and higher wages. Amenity-related wage and rent premiums can be calculated as location-fixed effects in hedonic regression equations that account for observable determinants of wages and rents. These wage and rent premiums are inputs into corresponding index values of QL and QB for each location, which then become key determinants in location choice modelling.

The empirical setting for our analysis is that of New Zealand. This country is of particular interest in the present context given that it has rich longitudinal microdata that can be derived from a set of integrated administrative datasets of individuals and firms, jointly referred to as the Integrated Data Infrastructure (IDI), collected and managed by Statistics New Zealand (Stats NZ).^3^ New Zealand is highly urbanised (with 83 per cent of the population living in urban areas) and has a high level of geographic mobility of skilled workers—including internationally.^4^ We use Stats NZ’s IDI and population census data to follow young people who graduate from an HEI and then reside in a New Zealand urban area in the early part of their careers.^5^ Every year about 125,000 students complete a formal qualification at an HEI—equivalent to 2.5% of New Zealand’s population of roughly 5 million.^6^ We focus only on HEI graduates who have remained within the country.We link their choices with QL, QB and some other features of the urban areas, as well as personal characteristics of the graduates, including their field of study. We account for the distance between their home (i.e. where the person went to school) and workplace, and between their HEI and workplace. The urban areas represent non-overlapping labour markets areas, i.e. there is very little if any commuting between them.

Based on Preston et al. (2018), Grimes et al. (2021) calculated wage and rent premia in 130 New Zealand urban areas using data from eight population censuses since 1976. Following Chen and Rosenthal (2008), these premia were converted into a QL value and QB value for each urban area and year. We analyse the determinants of the destination choices of tertiary graduates, given their HEI location, by means of the conditional logit regression model (McFadden 1974) and the mixed logit model (McFadden and Train 2000). We test whether students of different characteristics (viz. HEI type, field of study, HEI location, home location) locate in places that are regarded as good to live or good to do business. By incorporating prior school location we also test how the choice of work destination is affected by the pull of ‘home’.

We find that graduates are attracted to locate in places that have high quality production amenities. Creative Arts and Commerce graduates are relatively more likely to locate in places that are attractive to business, consistent with a symbiosis between bohemians and business (Florida 2002), and where entrepreneurship is thriving due to an abundance of human capital (Qian et al. 2013). Such working conditions are found in New Zealand most ubiquitously in the Auckland metropolitan area, which accounts for more than a third of the country’s population, and also in the capital city, Wellington. Hospitality and Personal Services graduates appear to be more drawn than other graduates to places with high consumption amenities. We conclude that places can leverage their existing (production or consumption) amenity strengths to act as drawcards to recent graduates, consistent with the principle of comparative advantage. We also see a strong pull of home and of the HEI location over the first 4 years of graduates’ working life.

The next section briefly reviews key literature on choice of location for tertiary education and for the first job. Features of our data and our estimation strategy are described in Sect. 3. Results are presented in Sect. 4, while conclusions and policy implications are discussed in Sect. 5.

## Key findings from the literature on location choice of recent graduates

Given that modern theories of regional growth assign considerable importance to education of the population as a driver of long-run growth (e.g. Mellander and Florida 2021; Glaeser et al. 1995), a large literature has emerged during the last two decades concerning two pivotal decisions in the life of young people with academic aptitude: firstly, where to study given the home location, personal characteristics, and what the available universities have to offer; and, secondly, where to work upon graduation. A full review of this literature is beyond the scope of this paper, but can be found in, e.g. Grimes et al. (2020). In this section, we cite key findings in the literatures on choice of location to study and the choice of where to work upon graduation. In our empirical work, we focus primarily on the latter, but we take the location of schooling into account as well. This allows us to test the ‘pull’ of home in the choice of where to work.

Regarding the move from home to university, the universal gravity law of migration (e.g. Poot et al. 2016) is present in a student’s selection of study location. Distance is a strong deterrent: closer HEIs are preferred over those further away (Sá et al. 2004). Students are also attracted to larger agglomerations (Sá et al. 2006), possibly because of a greater choice of HEIs (Böckerman and Haapanen 2013) and because of greater post-study job opportunities (D’Agostino et al. 2019). On the other hand, high rents in such agglomerations are a deterrent (Sá et al. 2004) and students do like natural amenties (Dotzel 2017). Hence universities are often located in so-called escalator regions that are peripheral but pleasant places to study (e.g. Wielgoszewska 2018). Students appear to be more influenced by their personal networks than by the prestige of HEIs: students often like to attend the same university as their high school peers (Sá et al. 2006). University prestige is likely to matter more in countries where the quality of HEIs varies a lot, with prestigous universities attracting students from wealthier backgrounds (Ro et al. 2021; Walsh et al. 2019).

During the last decade, a literature has emerged on the destinations of university graduates, and the impact of university-to-job transition, with papers in edited volumes such as Faggian et al. (2017) and Corcoran and Faggian (2017) being representative of the kind of studies undertaken. Corcoran and Faggian (2017) note that graduates have high geographical mobility, particularly in the first few years after graduating. The determinants of the propensity to move can be classified under three main headings: *social* (personal and family background, networks), *spatial* (push and pull factors of the home, university and potential employment destination regions) and *professional* (level and field of study, academic performance). Faggian et al. (2017) emphasise the importance of drawing on longitudinal data to better capture the spatial mobility from school through university to the labour market.

The vast majority of graduates are likely to be retained by major cities and thereby contribute to the increasing concentration of university graduates in metropolitan areas (e.g. Costa and Kahn 2000 in the USA; Ahlin et al. 2014 in Sweden; Corcoran et al. 2010 in Australia). Social networks are important, both professionally and personally (Teichert et al. 2020; Haapanen and Tervo, 2012). As in the case of the choice of university, distance plays an important role in the choice of the location of first employment too. Many studies find that the locations of parents and friends matter (e.g. Dahl and Sorenson 2010; Huttunen et al. 2018; Kaplan et al. 2016). In some cases family ties can be strong enough to induce graduates to return to peripheral regions (Crescenzi et al. 2017) but such graduates are likely to be those with poorer academic performance (Marinelli 2013). Study excellence and fields of study matter in post-graduation mobility (Haussen and Uebelmesser 2018). The more specialised are willing to move longer distances (Brown and Scott 2012). Amenities matter less after graduation (Gottlieb and Joseph 2006) although amenties such as sunshine and restaurants continue to appeal to the highly skilled who are able to secure high incomes (Buch et al. 2017). Gender (e.g. Haley 2018) and ethnicity (Zwysen and Longhi 2018) may matter too.

There is a much smaller literature that directly links the choice of school to HEI with that of HEI to first job. It is plausible that students select a place of study with future employment opportunities in mind (Abreu et al. 2014). Faggian and McCann (2009) find that the distance travelled from domicile to university in England is on average greater than the distance travelled between the university and the first job. Again, the attraction of large agglomerations for university students and graduates is very clear (see, e.g. Dutch evidence by Kooiman et al. 2018; Venhorst 2013). Ahlin et al. (2018) find that graduates in Sweden with better high school grades and from families with a strong educational background are more likely to start their labour market careers in urban regions, even if they grew up and went to high school in rural regions. Returning to the home region is often driven by personal, not economic reasons (Bjerke and Mellander 2017). Liu et al. (2017) find that the choice of university in China is predominantly driven by the spatial distribution of HEIs, but with preference for the national key universities, irrespective of distance. The subsequent distribution of university graduates in China is primarily driven by regional differences in wages. Similar evidence for South Korea is found by Ma et al. (2017).

## Description of data and estimation strategy

### Data

We use Stats NZ’s IDI, which includes both administrative and census data, to identify graduates and map their movements over time. We calculate urban quality of life (QL) and business (QB) indicators that can be obtained by means of 2006 and 2013 census data (in both cases the census was held in March). To match domestic graduates with economic conditions as they were at the time of deciding on their first job, our sample of graduates comprises all those who completed a qualification by the end of 2005, or by the end of 2012, and who satisfy certain criteria.^7^ Using education data, we identify the location of their HEI, which must have been attended intramurally. We also observe their high school, and therefore, ‘home’ location. We trace a graduate’s location 2 and 4 years after graduation and identify whether this was an overseas destination, and if not, in which urban area of New Zealand they chose to live (very few moved to rural areas, as shown below). We choose 2 years as our initial post-HEI destination since many students travel in their first year after graduation; and we choose 4 years as it allows for some initial sorting of job and location choices by graduates following their graduation and first job.

**Fig. 1:**
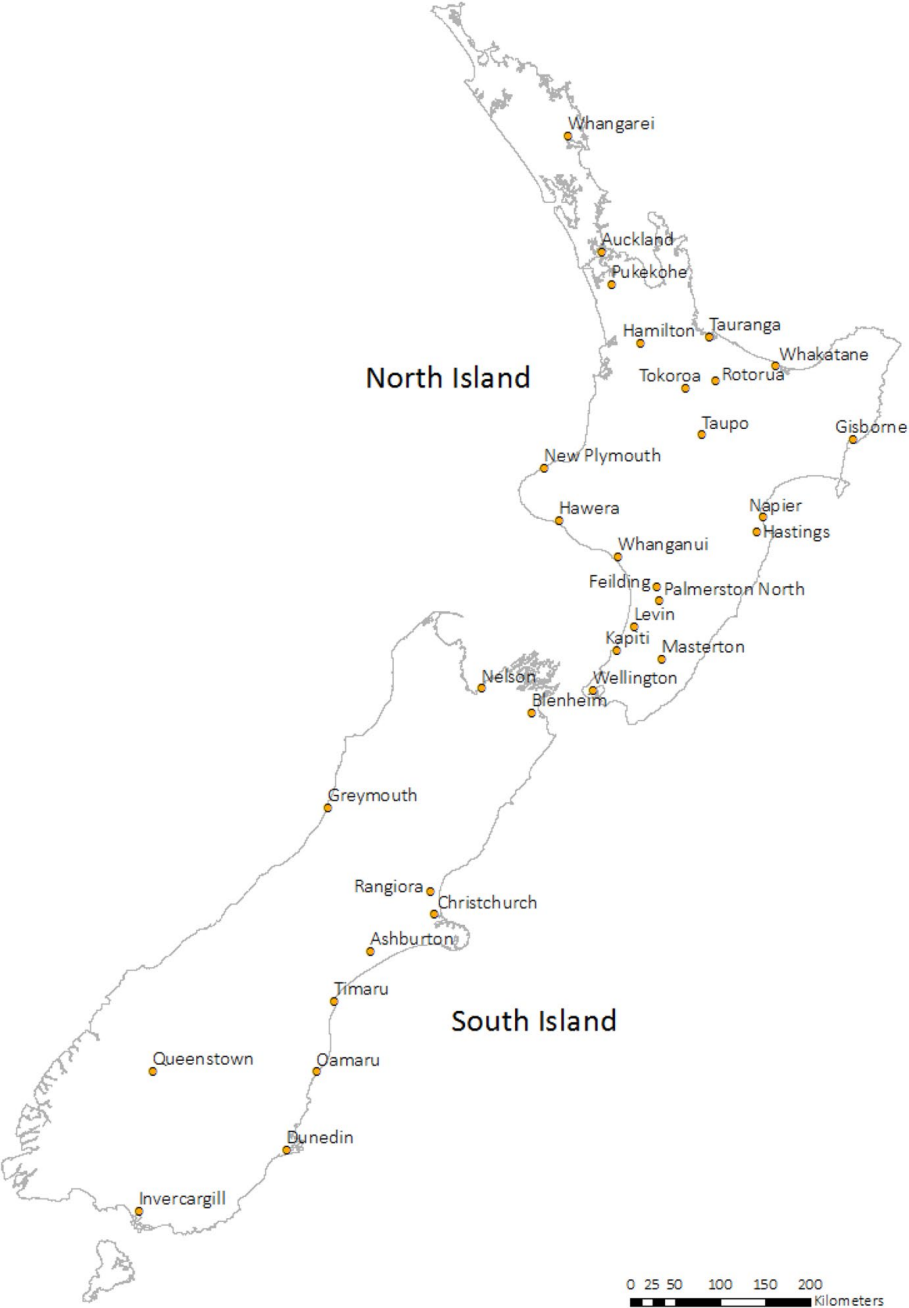
Map of New Zealand urban areas

We restrict our estimation sample to all individuals who are observed to live in one of 31 urban areas of New Zealand for all locations of interest: home, tertiary institute, and post-graduation destination 2 and 4 years after graduation.^8^ The map in Fig. 1 shows these urban areas.^9^ As noted in the introduction, these urban areas may be interpreted as non-overlapping labour markets and their boundaries have been stable over time (Newell and Perry 2005). They range in population from approximately 10,000 (Greymouth) to 1.3 million (Auckland). The Appendix lists the location of the HEIs. The top panel of Table 1 presents the total number of graduating students in 2005 and 2012, respectively, for whom we know their home location, HEI location, and location 2 and 4 years later.^10^ We observe that the majority of exclusions, especially for 4 years after graduating, occur due to international migration. There are also some exclusions due to rural areas being either the home, HEI, or destination location.^11^

**Table 1:** Sample composition (number of graduates)

Graduation year	2005		2012	
Years after graduation	2	4	2	4
*Full sample*	10,485	10,485	17,436	17,436
Rural locations	1203	1164	2664	2478
International destination	1608	2715	2229	3402
*Final sample*	7674	6606	12,543	11,556
Final sample comprises				
Non migrants	5016	4209	7650	6756
Late migrants	351	498	408	753
Return migrants	480	549	1236	1308
Repeat migrants	198	387	381	837
Tertiary stayers	1629	957	2868	1899

*Non-migrants* study in the home region and stay there upon graduation; *late migrants* study in the home region and then migrate elsewhere; *return migrants* move to the study region and then return home; *repeat migrants* move to the study region then move away from it, but not back home; *tertiary stayers* move to the study region and stay there upon graduation. See also Faggian et al. (2006). Any discrepancies in totals are due to random rounding to multiples of three. ‘Rural locations’ includes students whose Home, HEI or Destination is rural

The second panel of Table 1 outlines the composition of our final sample in terms of the categories proposed by Faggian et al. (2006). For each of 2 and 4 years after graduation, the largest category is *non-migrants*, i.e. students who do not leave the home region either to study, or after graduation. This group comprises approximately 60% of the final sample (for both 2 and 4 years after graduation). The high level of urbanisation in New Zealand and the relative concentration of population and graduates in large urban agglomerations contribute to the relatively large percentage of non-migrants. Only a small proportion of students study in their home region and then migrate within New Zealand (i.e. *late migrants*). Approximately two-thirds of students in our sample study in the ‘home’ urban area.

Of the students who study outside the home region (i.e. *return migrants*, *repeat migrants* and *tertiary stayers*), 66% stay in the HEI region 2 years after graduation, falling to 48% as the time from graduation extends to 4 years. A sizeable proportion of those who studied away from home (25% after 2 years and 31% after 4 years) returns to the home region, emphasising the strong pull of ‘home’.

A potential concern for our analysis is any bias that the selection criteria may introduce. A demographic breakdown of the student samples is provided in Table 2. The table also provides breakdowns of the fields of study [using the NZ Standard Classification of Education (NZSCED)] of graduates across all HEIs. There are 11 fields of study, plus a ‘mixed’ qualification category (2.8% of total). With few exceptions, such as health and architecture, graduates in each field can be found across all 29 HEIs.

**Table 2:** Demographic characteristics of samples

	Full sample (%)	International movers (%)	Rural movers (%)	Final sample (%)
*Student's HEI type*				
University	75.1	84.3	67.4	73.8
Polytechnic	24.9	15.7	32.7	26.2
*Gender*				
Male	41.6	40.9	36.6	42.9
Female	58.4	59.1	63.5	57.1
*Median age at graduation*				
Field of study				
Natural and physical sciences	12.6	13.5	12.1	12.4
Information technology	2.5	1.2	2.2	3.1
Engineering and related technologies	5.9	6.3	4.8	5.9
Architecture and building	3.3	2.4	3.9	3.6
Agriculture, environmental and related	1.3	1.0	4.2	0.7
Health	11.5	11.6	12.1	11.4
Education	5.5	4.5	7.8	5.2
Management and commerce	18.0	19.5	14.8	18.3
Society and culture	21.5	22.7	20.7	21.2
Creative arts	12.6	13.0	11.5	12.6
Food, hospitality and personal services	2.5	2.1	2.9	2.5
Mixed field programmes	2.8	2.2	2.8	3.0

Any discrepancies in totals are due to random rounding. The observations in the final sample are those of graduates observed four years after graduation (2005 and 2012 graduation cohorts combined)

We observe that the demographic characteristics of the graduates in the final sample are consistent with the characteristics of the full graduating sample. In some instances, we find that the final sample demographics reflect those of the full sample because of offsetting characteristics of the two excluded groups (i.e. those who move internationally and those who move rurally). For instance, university graduates are more likely to move internationally than are polytechnic students while the latter are more likely to move rurally. Similarly, natural and physical science graduates are over-represented in international movements and are under-represented in rural movements. By contrast, agricultural (and related) students are under-represented in international movements and strongly over-represented in rural movements.^12^

The QL and QB measures that we use reflect the consumption and production amenities, respectively, that are available in each location. We derive these measures following the approaches of Roback (1982), Gabriel and Rosenthal (2004) and Chen and Rosenthal (2008). Details can be found in Grimes et al. (2020). The value of consumption amenities of a location (i.e. QL) can be proxied by a function of local rents minus local wages. Intuitively, within a spatial equilibrium framework (in which people can shift location to maximise their utility), a location with high rents but low wages must have consumption amenities that make it a nice place to live; otherwise people would move elsewhere and newcomers would not arrive. For instance, *ceteris paribus*, a sunny coastal location can pay lower wages and/or charge higher rents relative to a rainy, inland location.

Similarly, production amenities of a location (i.e. QB) can be proxied by a function of local rents plus local wages. Intuitively, a location with high rents and high wages must have highly productive amenities that boost firms’ productivity otherwise firms would not locate in such a high cost location. Typically, cities with large populations experience agglomeration economies that enable firms to pay both high wages and high rents; yet many firms still choose to locate in these expensive locations because of the productivity benefits of doing so.^13^

**Fig. 2:**
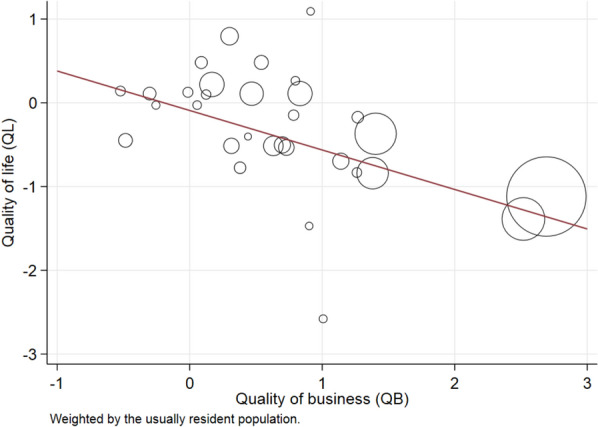
QL and QB values, 2013

QL & QB measures are standardised to have a mean of zero and standard deviation of one in the pooled data for 130 urban areas and all eight censuses between 1976 and 2013. Figure 2 depicts the 2013 values of QL and QB for the 31 urban areas in our study, where the size of each circle is proportional to population size. Two features are immediately apparent from Fig. 2. First, there is a strong negative correlation between locations’ QL and QB values (the unweighted Pearson correlation coefficient *r* = − 0.49 for 2013).^14^ Second, locations with larger populations tend to be more productive (i.e. to have high QB) but to have lower QL. The higher QB for larger places is consistent with agglomeration economies in those locations (Maré and Graham 2013). Their lower QL is consistent with a separate measure of quality of life derived from subjective well-being data. For instance, Morrison (2011) shows that residents in rural locations and smaller towns in New Zealand record higher levels of life satisfaction than do residents of large cities such as Auckland.

Table 3 presents the mean and standard deviation of quality of life and quality of business for each of the four event history locations, with a split by institution type (the data are pooled across 2005 and 2012 graduates). We observe that university students grow up in slightly lower quality of life locations compared with polytechnic students. They also tend to grow up in places which are better for business. These outcomes likely reflect a greater prevalence of university students coming from the larger cities relative to polytechnic students. When comparing Home, HEI and Year 4 Destination, we observe that university students become even more concentrated in places with high quality of business. Polytechnic students similarly gravitate towards higher QB places to study but then experience no further progression (on average) in QB following study. This may be driven by the different opportunities available to different types of students once they have completed their qualifications. Given that polytechnic students include those studying for trade qualifications, they are more likely to have suitable employment opportunities in the smaller New Zealand towns compared to university students who typically rely on the larger cities for work (Apatov and Grimes 2019).

**Table 3:** Quality of life and quality of business by location (2005 and 2012 graduates pooled)

Location	Quality of life (QL)			Quality of business (QB)		
	All	University	Polytechnic	All	University	Polytechnic
Home	− 0.856	− 0.883	− 0.772	1.937	2.000	1.743
	(0.649)	(0.644)	(0.658)	(1.102)	(1.094)	(1.105)
HEI	− 0.921	− 0.950	− 0.830	2.081	2.153	1.856
	(0.618)	(0.617)	(0.611)	(1.008)	(0.986)	(1.043)
Dest. Year 2	− 0.893	− 0.923	− 0.800	2.027	2.099	1.805
	(0.642)	(0.637)	(0.650)	(1.033)	(1.011)	(1.067)
Dest. Year 4	− 0.952	− 0.992	− 0.836	2.120	2.207	1.867
	(0.614)	(0.602)	(0.635)	(0.997)	(0.964)	(1.047)

For each location considered, graduates are located in one of 31 main and secondary urban areas (see Fig. 1). Standard deviation in parenthesis. QL & QB measures are standardised to have a mean of zero and standard deviation of one across 130 urban areas (that include minor urban areas) and all available census years (1976–2013)

University students tend to migrate over time to areas with lower quality of life. This trend is consistent over time, except for the second year after graduation, suggesting that some individuals may be migrating home temporarily.

**Fig. 3:**
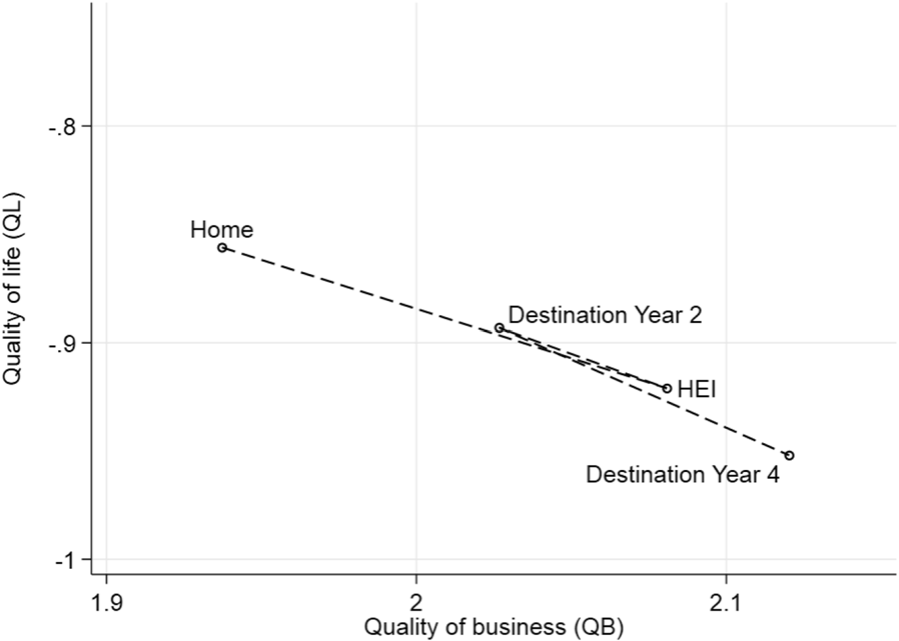
Mean quality of life and business by location (all HEI graduates)

Figure 3 presents the mean QL and QB measures for the final sample by life course stage. We observe the transition, discussed above, towards a higher quality of business location over time, with a brief period of lower QB (and higher QL) 2 years after graduating. The average quality of life decreases by 0.1 of a standard deviation, whereas quality of business increases by approximately 0.2 of a standard deviation between home and fourth year destinations.

**Fig. 4:**
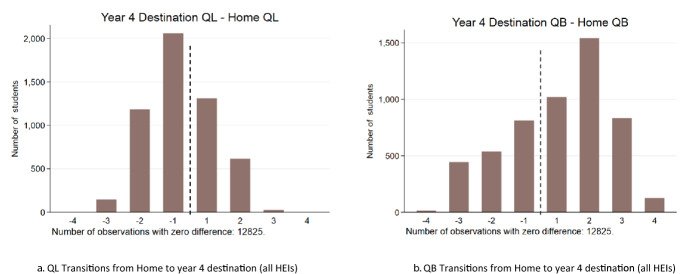
QL and QB transitions from Home to Year 4 destination (all HEIs). *Notes*: The zero category is omitted so that ‘movers’ can be seen clearly. The ‘− 1’ category is defined as [− 1, 0) while the ‘1’ category is (0,1], and similarly for the other categories in the graph

Figure 4a and b shows the distribution of the difference between home and year 4 destination locations in terms of QL and QB, respectively. For these figures, the zero category—being the category where individuals stay in their home location—is omitted so that the ‘movers’ can be seen more clearly. More than half of the individuals who experience a change are observed to lower their quality of life measure from home to destination, whereas close to two-thirds are observed to increase their quality of business measure. The magnitude of the changes indicates that a relatively small sacrifice in QL does, on average, correspond to a material increase in QB for those whose year 4 destination is not home.

**Fig. 5:**
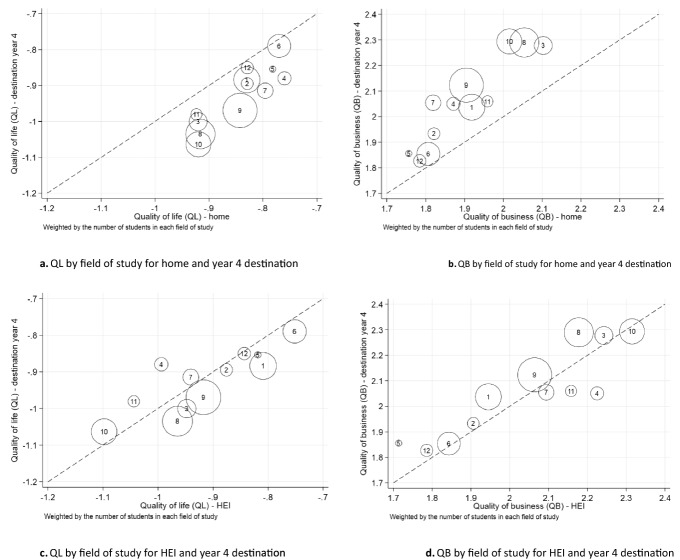
QL and QB at the home, HEI and Year 4 destinations by field of study (all HEIs). *Notes*: The circle areas are proportional to the total number of HEI graduates in each field of study, 2005 and 2012 combined. The fields of study are: (1) Natural and Physical Sciences (2) Information Technology (3) Engineering and Related Technologies (4) Architecture and Building (5) Agriculture, Environmental and Related Studies (6) Health (7) Education (8) Management and Commerce (9) Society and Culture (10) Creative Arts (11) Food, Hospitality and Personal Services (12) Mixed Field Programmes

Among the fields of study, we again observe a trend of graduates sacrificing quality of life for quality of business, but with significant heterogeneity in outcomes across the fields of study. Figure 5a–d presents the differences in quality of life and business for home and year 4 destinations; and for the transition from HEI to year 4 destination. Across all fields of study, year 4 destination QL is on average less than home QL. We observe that management and commerce, creative arts, food, hospitality and personal services and engineering and technology graduates have the lowest home QL. All of these, except food, hospitality and personal services graduates, congregate on average in higher quality of business destinations. Agricultural and education graduates originate from areas with high home QL and end up in destination year 4 in areas with low QB, although it may well be the case that these locations are specifically attractive to agricultural business activities.

The contrast between HEI QL and QB compared with the corresponding values in year 4 is much less than that between home QL and QB compared with year 4. Figure 5c and d shows heterogeneity in QL and QB change from HEI location to year 4 location across fields of study.

### Estimation strategy

We model the choice of location 4 years after graduation. We concentrate on the 4 year gap since it allows students to take some time for ‘trial and error’ in finding an appropriate location, as observed in the preceding figures of movements 2 and 4 years after graduation. We start with McFadden’s (1974) conditional logit model and extend this with the mixed logit model which allows coefficients to vary across individuals (McFadden and Train 2000). Graduates choose one of 31 urban areas. In the conditional logit model, the utility derived by graduate *i*, from alternative *j,* is given by:

$$U_{ij} = \beta^{\prime}x_{ij} + e_{ij}$$

where $\beta^{\prime}$ represents a vector of coefficients, $x_{ij}$ is a vector of observed location attributes which may vary by individual (for example, the distance from a potential year 4 destination to the individual’s home location), and $e_{ij}$ is a random error term which is assumed to be independent and identically distributed as a type I extreme value. Calculation of the probability that an individual selects alternative *j* relative to base *k* yields the conditional logit formula.

In our full specification, the vector of attributes ($x_{ij}$) for each of the 31 destinations (‘Dest.’) comprises^15^:

**Table:** 

Dest. QL:	quality of life (QL) in dest. location;
Dest. QB:	quality of business (QB) in dest. location;
Dest. ln(population):	logarithm of population of dest. location;
Dest. Δln(population):	annual population growth over prior intercensal period;
Dest. to home ln(distance):	logarithm of distance from dest. to home location;
Dest. to HEI ln(distance):	logarithm of distance from dest. to HEI location;
Dest. is home:	dummy variable (= 1 if dest. is the same as home location);
Dest. is HEI:	dummy variable (= 1 if dest. is the same as HEI location);
Dest. on home island:	dummy variable (= 1 if dest. is on same island as home location)^16^;
Dest. on HEI island:	dummy variable (= 1 if dest. is on same island as HEI location)

As discussed, QL and QB are included to proxy for all amenities that enhance consumption and production, respectively. An advantage of this modelling approach is that we do not have to choose which specific amenities (e.g. sunshine, coast, airport) to include. Population is included because QB and QL are correlated with city size, so inclusion of population enables us to test whether any estimated QB and QL effects hold once we control for city size. Additionallty, it is possible that students are attracted to growing places (e.g. for greater job opportunities) or else are deterred from such cities which may have housing shortages or location mis-matches within the city,^17^ so we include prior population growth to control for such influences.

We include six spatial terms in our estimation framework. We hypothesise that recent graduates are more likely to locate in their HEI or home location than other locations, and are more likely to locate on the same island as their home or HEI locations, given past findings that a shift in island is viewed as being costly (Preston et al. 2018).

The validity of a conditional logit approach rests upon the assumption of independence of irrelevant alternatives (IIA), which in the context of location choice may be unrealistic as unobservable characteristics likely influence preference for locations (Grimes et al. 2017). The mixed logit model allows coefficients to vary across individuals and is effectively an extension of the conditional logit model (Hole 2007). We assume a multivariate normal distribution for the random parameters so that the distribution parameters to be estimated are the means and standard deviations of each random coefficient.

To estimate a mixed logit model, simulation is required and this is computationally taxing. Given this computational challenge, and that our focus is on the quality of life and quality of business of the locations, we allow only the QL and QB coefficients to be random across individuals; the standard deviation of the coefficients are denoted ‘*SD Dest. QL*’ and ‘*SD Dest. QB*’, respectively.

When pooling students across all HEIs, we include a specification that contains interaction terms between each of the QL and QB measures and a dummy variable for polytechnic students to identify any differing behaviour by students according to institution type.Additionally, we assume full heterogeneity and run regressions separately for university students and for polytechnic students. We also test a specification that interacts a completion year dummy with all variables to test if our pooling across 2005 and 2012 cohorts is acceptable, and one that tests for differences in location preferences by gender. Neither specification indicates material differences by year of completion or by gender, and hence are not reported here.

## Results

Table 4 reports the conditional logit and mixed logit regression coefficients for student movements from HEI to their destination 4 years after graduation. Column (1) reports the conditional logit estimates for a simple model that contains just population, population growth, QL and QB. Students are observed to be attracted to places with high population and high quality of business. Quality of life has no significant effect while students are deterred from moving to places with high recent population growth.

**Table 4:** Pooled students—destination year 4

	(1)^a^	(2)	(3)^b^	(4)	(5)
Dest. ln(population)	1.115***	1.148***	0.616***	0.663***	0.665***
	(0.016)	(0.018)	(0.022)	(0.023)	(0.023)
Dest. *Δ*ln(population)	− 22.405***	− 21.649***	− 22.090***	− 20.825***	− 21.115***
	(1.694)	(1.706)	(2.608)	(2.735)	(2.741)
Dest. QL	0.048	− 0.050	0.348***	0.220***	0.250***
	(0.033)	(0.034)	(0.045)	(0.042)	(0.047)
SD Dest. QL		0.472***		0.721***	0.722***
		(0.047)		(0.035)	(0.035)
Dest. QB	0.218***	0.157***	0.515***	0.434***	0.494***
	(0.035)	(0.035)	(0.045)	(0.045)	(0.047)
SD Dest. QB		0.007*		0.007	0.007
		(0.004)		(0.010)	(0.010)
Dest. to home ln(distance)			− 0.085***	− 0.087***	− 0.087***
			(0.006)	(0.006)	(0.006)
Dest. to HEI ln(distance)			− 0.060***	− 0.060***	− 0.059***
			(0.005)	(0.006)	(0.006)
Dest. is home			2.544***	2.613***	2.609***
			(0.039)	(0.041)	(0.041)
Dest. is HEI			2.443***	2.524***	2.506***
			(0.035)	(0.037)	(0.037)
Dest. on home island			0.073*	0.121***	0.114***
			(0.038)	(0.039)	(0.040)
Dest. on HEI island			0.471***	0.552***	0.568***
			(0.039)	(0.041)	(0.041)
Dest. QL * Polytechnic					− 0.090
					(0.073)
Dest. QB * Polytechnic					− 0.231***
					(0.041)
Observations	18,162	18,162	18,162	18,162	18,162
Log likelihood	− 35,973.2	− 35,962.9	− 16,551.3	− 16,495.9	− 16,473.7

Model 1 and 3 are conditional logit models; models 2, 4 and 5 are mixed logit models (regression coefficients reported in each case). Huber–White robust standard errors in parentheses

^*^p < 0.1, **p < 0.05, ***p < 0.01

^a^Pseudo *R*-squared = 0.423

^b^Pseudo *R*-squared = 0.735

In column (2), we use the mixed logit model to estimate the same specification with random variation for the QL and QB terms. The standard deviation term for QL is significant indicating that students respond in a heterogeneous manner to consumption amenities. There is little heterogeneity apparent with regard to production amenities.

Columns (3) and (4) present preferred specifications, reporting conditional and mixed logit estimates, respectively, with the spatial terms added. Column (5) extends the mixed logit specification to test if polytechnic students (represented using the interactive ‘*Polytechnic*’ dummy variable) respond differently to university students with respect to QL and QB.

The results in columns (3) to (5) again indicate that graduates locate in places that have beneficial quality of business. This effect is weaker for polytechnic students than for university students. With the spatial terms added, quality of life is also found to be an attractor, with significant heterogeneity in response. By contrast, there is no significant heterogeneity in response to QB. The interaction terms indicate that the effect on student location choice of a one standard deviation change in QB relative to a one standard deviation change in QL is greater for university students than for polytechnic students. The importance of QB relative to QL for university students is consistent with university graduates choosing high production amenity places at the outset of their careers even if these places have lower quality of life.

In addition to these estimated responses to our main variables, we see that students are more likely to locate in larger places, in their home and HEI locations (and islands), and in places that are close to their HEI and to their home. These spatial responses are all as anticipated.

One possibly surprising result across all specifications is that recent population growth acts as a deterrent for student location choice. Places with recent fast population growth may face a temporary housing shortage which pushes up rents temporarily and/or forces new graduates to locate in unfavourable areas within a city. By construction, temporarily high rents will result in high values for both the QL and QB variables but this may not accurately reflect longer term equlibrium valuations placed on consumption and production amenities, which are what our QL and QB variables are designed to represent. The inclusion of lagged population growth potentially acts as a correction for this dynamic effect associated with temporarily high rents.

The significance of the polytechnic interaction term for QB in Table 4 raises the possibility that the responses of students from universities and polytechnics to other variables may also differ across institution type. In Table 5, we present separate estimates for university and for polytechnic students corresponding to columns (3) and (4) of Table 4. University students show greater responsiveness to both QL and QB than do polytechnic students. Students from both types of institution are drawn to locate in larger places, but this effect is more strongly observed for university students, who are also more responsive to recent population growth.

**Table 5:** University and polytechnic students—destination year 4

	University students		Polytechnic students	
	(1)^a^	(2)	(3)^b^	(4)
Dest. ln(population)	0.679***	0.720***	0.471***	0.528***
	(0.026)	(0.027)	(0.042)	(0.043)
Dest. *Δ*ln(population)	− 24.172***	− 23.112***	− 14.476***	− 13.186**
	(2.985)	(3.109)	(5.461)	(5.769)
Dest. QL	0.408***	0.258***	0.182**	0.147*
	(0.053)	(0.052)	(0.086)	(0.077)
SD Dest. QL		0.679***		0.772***
		(0.045)		(0.061)
Dest. QB	0.533***	0.446***	0.419***	0.369***
	(0.053)	(0.054)	(0.088)	(0.088)
SD Dest. QB		0.008		− 0.010
		(0.011)		(0.035)
Dest. to home ln(distance)	− 0.090***	− 0.091***	− 0.060***	− 0.061***
	(0.006)	(0.007)	(0.013)	(0.014)
Dest. to HEI ln(distance)	− 0.061***	− 0.060***	− 0.057***	− 0.063***
	(0.006)	(0.006)	(0.012)	(0.013)
Dest. is home	2.442***	2.496***	2.844***	2.926***
	(0.046)	(0.048)	(0.078)	(0.080)
Dest. is HEI	2.465***	2.525***	2.216***	2.339***
	(0.041)	(0.043)	(0.078)	(0.082)
Dest. on home island	0.072*	0.115***	0.093	0.147
	(0.041)	(0.043)	(0.098)	(0.102)
Dest. on HEI island	0.406***	0.487***	0.704***	0.778***
	(0.043)	(0.045)	(0.095)	(0.100)
Observations	13,512	13,512	4653	4653
Log likelihood	− 12,657.4	− 12,628.0	− 3817.1	− 3796.1

Model 1 and 3 are conditional logit models; models 2 and 4 are mixed logit models (regression coefficients reported in each case). Huber–White robust standard errors in parentheses

^*^p < 0.1, **p < 0.05, ***p < 0.01

^a^Pseudo *R*-squared = 0.727

^b^Pseudo *R*-squared = 0.761

As well as responses to QL and QB differing between university and polytechnic students, it is quite possible that responses to consumption and production amenities differ by field of study. To explore this potential heterogeneity in response, we estimate specifications that allow for different responses for students from different fields of study (FOS). We base these estimates on the specification in column (2) of Table 5, with two added terms in which we interact a specific FOS with each of QL and QB. Hence these estimations are done with the sample of university graduates only (*n* = 13,512). Results are reported in Table 6. Each row of Table 6 reports results for the impacts of QL and QB for a particular (i.e. separate) FOS equation.

**Table 6:** Effects of QL and QB by field of study (FOS), university students only—destination year 4

Field of study (FOS)	(1)	(2)	(3)	(4)
	QL*FOS	QB*FOS	QL + QL*FOS	QB + QB*FOS
Sciences	0.083	− 0.073	0.289***	0.372***
	(0.078)	(0.047)	(0.074)	(0.058)
IT	− 0.203	− 0.107	0.024	0.330***
	(0.168)	(0.100)	(0.168)	(0.107)
Engineering	− 0.122	− 0.010	0.106	0.425***
	(0.146)	(0.085)	(0.145)	(0.092)
Architecture	0.290	0.046	0.501*	0.478***
	(0.183)	(0.109)	(0.182)	(0.114)
Agriculture/environmental	− 0.173	− 0.332**	0.050	0.106
	(0.298)	(0.150)	(0.298)	(0.155)
Health	− 0.132	− 0.325***	0.109	0.152**
	(0.086)	(0.051)	(0.082)	(0.063)
Education	0.040	− 0.066	0.259*	0.371***
	(0.156)	(0.091)	(0.155)	(0.098)
Commerce	0.043	0.314***	0.262***	0.705***
	(0.091)	(0.054)	(0.089)	(0.065)
Society	− 0.108	0.012	0.137*	0.441***
	(0.079)	(0.045)	(0.076)	(0.058)
Creative Arts	0.152	0.199***	0.356***	0.609***
	(0.116)	(0.067)	(0.115)	(0.077)
Hospitality	0.217	− 0.090	0.433*	0.346**
	(0.246)	(0.130)	(0.245)	(0.136)
Mixed	0.321*	− 0.050	0.531***	0.383***
	(0.170)	(0.109)	(0.168)	(0.114)

Each row represents a separate equation in which a single FOS is entered along with the base equation [column (2) of Table 5]; each equation includes all variables in the base equation (not reported). QL*FOS & QB*FOS regression coefficients represent the difference in QL & QB for that FOS relative to all other fields. QL + QL*FOS and QB + QB*FOS is the linear combination of the base QL and QB regression coefficient and the FOS interaction term with QL or QB. Huber–White robust standard errors in parentheses

^*^p < 0.1, **p < 0.05, ***p < 0.01

The first two columns report the coefficient on the interaction term between each FOS and QL and QB, respectively. The interaction term indicates how the QL and QB responses for that FOS differ from the average for all other fields. Other than the ‘Mixed’ category, no FOS differs significantly from the others in terms of its reaction to QL.

With respect to QB, we find that graduates from two fields are less attracted to places with high quality of business than are other graduates. Health graduates are required throughout the country, so a high quality of business is not a particular drawcard for these students, and graduates in the Agriculture and Environmental fields are likely to situate in smaller communities (with lower QB) that service rural needs.

Commerce graduates are more likely than the average to locate in places with high quality of business, consistent with agglomeration economies for these graduates. Creative Arts graduates are also more likely to locate in such places, reflecting the types of synergies between business and the arts discussed by Florida (2002). The QB results for Commerce and Creative Arts graduates may well reflect the strong pulls of Auckland, the largest metropolitan area, and of Wellington, the capital city.

When we consider the combined coefficients that show the full effect for each of QL and QB (columns (3) and (4), respectively, of Table 6), we find that Quality of Life is an attractor (with *p* < 0.1) for graduates in the Sciences, Architecture, Education, Commerce, Society, Creative Arts, and Hospitality (plus Mixed). Quality of Business is an attractor for graduates from all fields of study other than Agriculture/Environment (with these graduates more likely to be attracted to places with business environments suited specifically to those fields). Consistent with our heterogeneity results in Tables 4 and 5, we therefore again observe greater heterogeneity of response with respect to QL than we do with respect to QB. Quality of Business is an almost ubiquitous attractor for graduates, whereas the location response to Quality of Life differs more markedly across fields of study.

We gain greater insights into the relative importance of consumption and production amenities by calculating the marginal effects of a one standard deviation change in each of QL and QB on the probability of locating in each city. The marginal effects are derived using our preferred (aggregated) specification, column (4) of Table 4. The calculation takes into account the heterogeneity associated with QL and QB and also takes account of each student’s own circumstances (e.g. distance of each city from the student’s home, HEI, etc.); it also accounts for the nonlinearity of the specification.

These calculations^18^ indicate that the point estimate for the overall QL marginal effect is, on average, slightly larger than that for QB. However, the effect of a change in QL on student location choice is not statistically significant at the 10% level in any of the urban areas, whereas the marginal effect of QB change is statistically significant at the 1% level in all urban areas. The QL result reflects the estimated heterogeneity in preferences with respect to consumption amenities. If students are locating on the basis of prospective jobs and incomes (i.e. with respect to QB) it is reasonable to expect that there will be little heterogeneity with respect to the effect of production amenities. By contrast, tastes with respect to consumption amenities differ widely across students. Thus a rise in QB is likely to have a similar effect on location choice for different types of students, whereas there is less predictability about whether any particular student will be attracted to a specific bundle of consumption amenities in different locations.

## Conclusions

We analyse the within-country location choice of HEI graduates in New Zealand following their studies. Specifically, we focus on the movements of graduates whose home, HEI and destination 2 and 4 years after graduation are each within 31 urban areas of New Zealand. The estimation sample comprises over 18,000 students out of two graduating cohorts.

We bring together the literatures on graduate location choice with that on locational amenity values. These locational amenities are measured using the ‘quality of life’ (QL) and ‘quality of business’ (QB) metrics arising from the work of Roback (1982), Gabriel and Rosenthal (2004) and Chen and Rosenthal (2008). A place with high quality of life has beneficial consumption amenities, so residents are prepared to accept high rents and/or low wages. A place with high quality of business has beneficial production amenities, so firms are prepared to pay high rents and high wages.

At a descriptive level, we find that students tend to move from home to HEI to fourth year destination on a gradient of falling quality of life and rising quality of business. The negative correlation between the two quality measures reflects the findings of Morrison (2011) and Preston et al. (2018) that larger cities have lower quality of life, perhaps because of congestion and lower disposable income after housing costs, while enjoying agglomeration benefits (Maré and Graham 2013).

The trajectory of graduate migration reflects one of a drift towards the larger settlements. One slight interruption to this pattern is that graduates tend to revert 2 years after graduation to lower QB and higher QL places relative to their HEI, before their longer term location choice favours places that are better for business. The direction of movement to a higher quality of business location from home to fourth year destination occurs, on average, for students across all fields of study, though the direction of movement differs between HEI and destination reflecting different skill demands in different places.

In modelling the relationship between graduates’ destination choices and locations’ QL and QB we confirm the positive association of graduate destination choice with the locational quality of business, with very little heterogeneity of response. We also find that a higher quality of life helps to attract graduates to a place, but the response to quality of life displays considerable heterogeneity across graduates. The effects of each type of amenity are stronger for university graduates than for those from polytechnics. We also find a strong pull of home for many students, plus a pull to remain in the chosen HEI destination, while a larger population acts as an attractor. By contrast, graduates are less likely to locate in places that have had recent high population growth, possibly reflecting temporary housing constraints.

Relative to other graduates, those with Management and Commerce qualifications are attracted to places with a high quality of business, while Creative Arts graduates are attracted both to places with high quality of business and high quality of life. Artistic graduates’ attraction to places with high QL may reflect the preferences of those who study in the creative arts. The attraction of both Creative Arts and Commerce graduates to places with high QB is consistent with the beneficial effects for cities that mix bohemian and business elements in large metropolitan areas (Florida 2002).

Our results for graduates can be contrasted with those of Grimes et al. (2021) who examined location choices of adults aged 25–54 years within New Zealand. New Zealand residents of this age-group are primarily drawn to places with high quality of life, while recent migrants to New Zealand are attracted to places with high quality of business. The location choices of recent graduates has a plausible consistency with the behaviour of recent international migrants. Both international migrants and graduates are at the outset of their working careers within New Zealand, and so quality of business is likely to be more important for these groups than it is for established workers. A pattern of locating early in life in places with high wages, even if they have low consumption amenities, is consistent with lifetime utility maximisation for those with a low rate of time preference (Grimes et al. 2017).

While our estimated impacts are based on associative relationships, the results may be useful for local decision-makers when it comes to planning for the demographic and skills composition of their local settlement. For instance, decisions that favour the strengthening of production amenities relative to consumption amenities are more likely to result in a higher proportion of Commerce and Management graduates than would policy decisions that favour consumption amenities. Thus local investment decisions regarding amenities will influence not only the number, but importantly also the type, of local graduate that is attracted.

## Appendix

See Table

**Table 7:** New Zealand higher education institutes

Higher education institute name	Type	Urban areas	EFTS 2012
Aoraki Polytechnic	Polytechnic	Timaru, Ashburton, Oamaru	1910
Bay of Plenty Polytechnic	Polytechnic	Tauranga	3040
Unitec New Zealand	Polytechnic	Auckland	10,335
Ara Institute of Canterbury	Polytechnic	Christchurch	5920
Eastern Institute of Technology	Polytechnic	Hastings, Gisborne	4285
Wellington Institute of Technology	Polytechnic	Wellington	4065
Universal College of Learning	Polytechnic	Palmerston North	3570
Manukau Institute of Technology	Polytechnic	Auckland	7525
Nelson Marlborough Institute of Technology	Polytechnic	Nelson, Blenheim	3090
Northland Polytechnic	Polytechnic	Whangarei	3310
Otago Polytechnic	Polytechnic	Dunedin	3705
Whitireia Community Polytechnic	Polytechnic	Wellington	4415
Southern Institute of Technology	Polytechnic	Invercargill	4410
Western Institute of Technology Taranaki	Polytechnic	New Plymouth	1955
Waiariki Institute of Technology	Polytechnic	Rotorua, Tokoroa, Whakatane, Taupo	3945
Waikato Institute of Technology	Polytechnic	Hamilton	5860
Open Polytechnic	Polytechnic	Wellington	6055
Tai Poutini Polytechnic	Polytechnic	Greymouth	2390
Te Wananga O Aotearoa	Wānanga	Hamilton	20,495
Te Wananga O Raukawa	Wānanga	Otaki (between Kapiti and Levin)	1335
Te Whare Wananga O Awanuiarangi	Wānanga	Whakatane	2905
University of Auckland	University	Auckland	32,600
University of Waikato	University	Hamilton	10,165
Massey University	University	Palmerston North, Wellington, Auckland	19,680
Victoria University of Wellington	University	Wellington	17,220
University of Canterbury	University	Christchurch	13,085
Lincoln University	University	Christchurch	3560
University of Otago	University	Dunedin	19,160
Auckland University of Technology	University	Auckland	18,765

This list refers to HEIs in 2012. Some have subsequently merged under new names. Some HEIs have campuses in multiple cities. The listed urban areas correspond to those shown in Fig. 1. EFTS: Equivalent full-time students

*Source*: https://www.educationcounts.govt.nz/statistics/tertiary-participation

7.

## Footnotes

1 While e-learning can be in principle a substitute for classroom learning, in practice most students continue to enrol in ‘bricks and mortar’ institutions. The evidence on the effectiveness of e-learning vis-à-vis classroom learning remains inconclusive (Bernard et al. 2004). Quasi-experimental evidence is now emerging from the COVID-19 pandemic that may shed more light on this (e.g. Zheng et al. 2021).

2 Manic (2019) provides evidence (in a European setting) that international emigration also triggers internal migration of graduates from rural to urban areas.

3 A description of the features and applications of the IDI can be found at https://www.stats.govt.nz/integrated-data/integrated-data-infrastructure/

4 Ten years after study, about a third of young domestic university graduates live abroad (Ministry of Education 2017).

5 We use the terms higher education institutions (HEIs) and tertiary institutions interchangeably. They include both universities and polytechnics. Polytechnics include institutes of technology and wānanga. A wānanga is a Māori-specific (i.e. indigenous) category of HEI. During the period covered by our data there were 8 universities and 21 polytechnics (including 3 wānanga) in New Zealand. The Appendix provides a list of HEIs with their locations and an indicator of HEI enrolment (the number of equivalent full-time students (EFTS) enrolled in 2012).

6 See https://www.educationcounts.govt.nz/statistics/achievement-and-attainment. Our sample of students, described in Sect. 3, accounts for only one eighth of these because the sample excludes students at the lowest qualification levels (1–3), those who studied parttime, and those who did not start study immediately after completing high school.

7 Qualifications must be New Zealand classification level 4 (post-school certificate) or above, completed intramurally, full-time, started no more than 1 year following the completion of high school, and must have involved contiguous study. The student must also be a domestic student and may not have enrolled in further full-time study within 4 years of completion of their 2005 or 2012 qualification. We include summer completions for each year. For example, a January 2006 completion is included as a 2005 completion.

8 We include individuals in the ‘2 year’ sample even if they are not observed in the fourth year after graduation, and include individuals in the ‘4 year’ sample even if they are not observed in the second year after graduation; the latter case may be due to the individual travelling overseas in the intervening period.

9 The 31 urban areas are those classified by Stats NZ as main and secondary urban areas. Napier and Hastings are adjacent cities which we aggregate to a single urban area given the commuter flows between them. We have chosen to leave two other borderline cases, Pukekohe (near Auckland) and Kapiti (near Wellington) as separate urban areas, reflecting lower commuter flows at the start of our sample.

10 The 2012 full sample in Table 1 is substantially larger than that for 2005 mostly because of improved coverage of home (school) location for the former group within the administrative data.

11 ‘Rural’ here refers to minor urban areas (small towns), genuinely rural areas, and unknown locations.

12 While Table 2 shows that the removal of rural and international movers from the sample does not greatly influence the representativeness of our sample of HEI graduates, the different groups of movers may exhibit heterogeneous mobility behaviour. The study of this heterogeneity is beyond the scope of the present paper. New Zealand has a diaspora equivalent to about one fifth of its population and the percentage among those with tertiary education is among the highest in the OECD (Dumont and Lemaître 2005). Alimi et al. (2019) model New Zealand’s gross internal migration flows jointly with international migration but this approach has yet to be extended to microeconometric choice modelling. The results reported in Sect. 4 are therefore conditional on the decision of graduates to remain in (or return to) New Zealand.

13 Preston et al. (2018) document patterns of consumption and production amenities across locations in New Zealand corresponding to QL and QB for those locations. They find that places with larger populations tend to have higher QB (but lower QL) while places with high QL tend to be sunny, dry, near a body of water (i.e. the sea or a lake), and close to tourism facilities. There is also some evidence that places with high QL tend to have relatively high shares of the workforce engaged in education and health.

14 Similarly, *r* = − 0.50 for 2006.

15 Each of QL, QB and ln(population) are contemporaneous; ln(population) growth is over the previous inter-censal period.

16 All our 31 urban areas are on either the North Island or the South Island of New Zealand.

17 Grimes and Hyland (2015) show that housing stock changes lag behind population changes in New Zealand cities, resulting in temporarily high housing costs in faster growing cities.

18 See Grimes et al. (2020) for detailed results for each urban area.

## Declarations

### Disclaimer

Access to the data used in this study was provided by Statistics New Zealand under conditions designed to give effect to the security and confidentiality provisions of the Statistics Act 1975. All frequency counts using Census data were subject to base three rounding in accordance with Statistics New Zealand’s release policy for census data. The views, opinions, findings and conclusions or recommendations expressed in this paper are strictly those of the authors and do not necessarily represent, and should not be reported as, those of the organisations at which the authors are employed.
